# HIV vaccine: it may take two to tango, but no party time yet

**DOI:** 10.1186/1742-4690-6-88

**Published:** 2009-10-09

**Authors:** Ben Berkhout, William A Paxton

**Affiliations:** 1 Laboratory of Experimental Virology, Department of Medical Microbiology, Center for Infectious Diseases and Immunology Amsterdam (CINIMA), Academic Medical Center (AMC), University of Amsterdam, the Netherlands

## Abstract

A press conference on Thursday September 24 in Bangkok, Thailand, released data that an experimental vaccine provided mild protection against HIV-1 infection. This is the first positive signal of any degree of vaccine efficacy in humans, more than a quarter-century after scientists discovered the virus that causes AIDS. The research was conducted by a team including Thai researchers, the U.S. Army and the U.S. National Institutes of Health. The RV144 Phase III clinical trial, which began in 2003, had been disparaged by many critics as a waste of time and money because each of the two components had been shown to produce no benefit as individual vaccines and because the scientific rationales behind the immunogens were just wrong. It was nevertheless speculated that using them together in the prime-boost scenario could be more effective, with the aim to induce heightened CD4^+ ^cellular immune responses against the viral Envelope protein. This optimism seems to have been validated. In fact, this would not be the first time that the discovery of an effective vaccine relied as much on serendipity as opposed to scientific rationale. On the other hand, many questions remain about the RV144 trial, and these issues will be addressed in this editorial.

## The press conference

The limited information provided by the trial sponsors in their press release is that 74 out of 8,198 volunteers who received placebo immunizations became infected with HIV-1 compared to 51 out of 8,917 volunteers who received the prime-boost vaccine. The results equate to a protective efficacy of a little over 31%, with a p value of less than 0.039, just below the widely accepted significance cutoff of 0.05. The vaccine had no effect on post-infection viral loads among the recipients who became infected.

This 31% value is below the 50% reduction rate defined as "unequivocal clinical benefit", and the margin of error is wide; so it does not suggest that the experimental vaccine should now be deployed for general use. On the other hand, it is enough to justify further research into deciphering the underlying mechanism which provides for the protection observed and - when the results will be sustained during the coming months - putting additional efforts into improving this approach.

## The vaccine components

The priming vaccine, called ALVAC and made by Sanofi Pasteur in Lyon, France, consists of a version of the canarypox viral vector that was engineered to contain synthetic versions of three HIV-1 genes that encode the Gag and Protease proteins of subtype B and a chimeric form of the Envelope protein (subtype E gp120 linked to a portion of the subtype B gp41 domain). This type of viral expression vector is roughly similar to the adenovirus vector that was tested by Merck in the STEP trial that failed to show protection [1]. Further study of the STEP results found a potentially increased risk of HIV-1 infection among vaccinated men who had high levels of pre-existing immunity against the vector. These viral vector based vaccines are designed to stimulate human immune cells to fight HIV-1.

The boosting antigen in AIDSVAX is a genetically engineered version of the gp120 Envelope protein, originally designed by the California biotech firm VaxGen. This bivalent vaccine contains equal concentrations of the subtype B and E gp120 antigen. Its purpose is to stimulate the production of antibodies that can neutralize HIV-1.

The antigens used in the vaccine come from the subtypes which are the most common forms in, respectively, North America and Europe, and in South-East Asia. Thus, there is a perfect match between this vaccine and the test location in Thailand. Both ALVAC and AIDSVAX vaccines consist of only pieces of HIV-1, not the whole virus, and therefore cannot cause AIDS and both are intended for those uninfected with HIV-1, to educate their immune systems to be able to fight off HIV-1 infection should they later be exposed to the virus.

The priming component of the RV144 trial, ALVAC, had been tested for safety in previous trials, but none of those trials was designed to see if it was efficacious [2]. The boosting component, AIDSVAX, had been the subject of a previous trial in Thailand, and also one in North America and Europe. Those two trials, intended to raise a traditional antibody-based response, were regarded at the time as the best hope for an effective vaccine, although they also met with critiques up front from the scientific community because the Envelope protein used adopts a conformation that raises predominantly the wrong, non-neutralizing antibodies. The trials were a bitter disappointment, showing no effect whatsoever, although AIDSVAX was shown to be safe [3].

## The specifics of the RV144 trial

A full vaccination course of the RV144 regime lasts six months and consists of four ALVAC shots and two AIDSVAX shots, with a 50% chance of this being a placebo rather than the vaccine. The adult volunteers between ages 18 and 30 were drawn from the general public, rather than specifically from groups at risk such as men having sex with men (MSM), recreational drug injectors, or commercial sex workers (CSW). This may explain in part the low number of persons that acquired HIV-1 during the 6 year follow-up. In fact, only 125 of the 17,115 participants became infected during the trial, which is in all likelihood due to effective HIV-1 prevention counseling and condom distribution. Indeed, this may turn out to be the biggest success story of this trial. With such low numbers, it would have been important to obtain another independent measure of vaccine efficacy. Vaccines that provide protection against virus infection usually also have an impact on the amount of virus that can be measured in the individuals that failed to be protected. Vaccine studies in the SIV-macaque model have also indicated that it is easier to obtain a therapeutic effect (reduction of viral load in established infection) than a truly prophylactic effect (protection against a new infection). Possibly the most surprising result of the RV144 study is that the vaccine appeared to provide mild protection while having no effect on the viral load in those who became infected. Such a scenario points to good induced mucosal responses that do not translate to control of viral replication following HIV-1 infection. This result, which provides for much of the excitement to the study, remains a big puzzle that should be addressed in follow-up experiments. However, for the moment an independent measure of vaccine efficacy is lacking.

The restrained enthusiasm seems appropriate in the absence of more detailed information on the study results. In particular, based on the limited amount of information provided, the statistical significance hangs on a very few cases of HIV-1 infection. For instance, the results could be skewed by subtle differences between the vaccine and control groups, in particular because the overall percentage of HIV-1 infection is relatively low in this study group. Because of the small numbers, the accidental overrepresentation of some individuals engaged in high-risk sexual activity or condom usage could have a big effect on the statistical significance reached. The RV144 study did not exclude high-risk people, and at baseline about one-fourth reported having high-risk behavior such as MSM or CSW. Male circumcision is also a factor associated with risk of HIV-1 transmission, and obviously the overrepresentation of circumcised or non-circumcised men in the two groups could confer some bias. Interestingly, male circumcision has proved to have a higher protection rate against HIV-1 transmission than reported with this vaccine. It is expected that more data will be presented at the annual AIDS Vaccine Conference in Paris (October 19-22, 2009), but how much information will be released remains to be seen.

## How to proceed

The trial has certainly posed more new questions than it has so far answered. Some of these issues can be addressed in follow-up analysis of the materials collected during the trial, but new clinical trials would seem required to address other issues. First of all, it would seem important to arrange an extended follow-up of the RV144 trial, if possible, to increase the numbers and to test for durable vaccine effects.

Follow-up laboratory work can address several issues. The key issue is to identify the correlates of protection. In other words, what mechanism or molecule correlates with the observed protection, and can this also explain why no effect is seen on the viral load in those individuals that became infected despite receiving the vaccine? This reminds us of the early progress on live-attenuated SIV variants in the macaque model, where good protection was obtained [4]. However, the correlates of protection were never clearly defined, and this track has lost momentum because these replicating vaccine viruses turned out not to be safe [5]. Another relevant issue is to dissect whether the observed protection shows specificity for the B or E subtype, which is possible because the ALVAC component does not have an identical B/E composition. In fact, such a difference may improve the statistical relevance of the study. Likewise, ethnic differences may also be relevant to consider [6]. Additionally, viral sequencing and comparison between infected vaccine and placebo recipients may provide insights into whether Envelope sequences have been selected to escape from vaccine pressure.

A major issue is that, in the absence of efficacy data on ALVAC as an individual vaccine, it will be difficult to dissect whether inclusion of AIDSVAX yielded any benefit. Possibly laboratory analysis can help to provide an answer, but otherwise new human trials will be required to address this issue. New trials would also seem required to determine whether similar results can be obtained in high-risk populations such as MSM, injecting drug users, and high-risk heterosexuals such as CSW.

It will require hard work to translate this landmark result into a true public health benefit. One criticism of the RV144 study design is that it does not match the situation in sub-Saharan Africa where two-thirds of the world's HIV-infected people live, and two-thirds of all new infections are happening. This continent, where HIV-1 originated from multiple zoonotic transfers from primates, has the greatest variety of HIV-1 subtypes, but subtypes B and E are not common there. However, it seems relatively straightforward to modify the RV144 components to match the subtypes that are more relevant at the global level, such as subtype A and C.

The results of the RV144 trial may indicate that we should not put all our money on basic science and ignore clinical science, as human experimentation has added value over what we can test in tubes and animal models. However, it seems critical to maintain the appropriate balance between basic vaccinology science and empirical clinical trials. At the same time, we should not make the mistake of focusing future vaccinology research exclusively on the components of the RV144 vaccine and thereby hamper the development of other innovative approaches. This seems particularly important because nobody can predict which vaccine direction will lead to an effective and safe vaccine that protects against infection. One lesson learned is not to throw away old vaccines too quickly, but this should not jeopardize the research pipeline towards novel vaccine approaches. Such alternative strategies range from the molecular manipulation of the viral Envelope protein to become a better immunogen [7-9], to the presentation of early HIV-1 regulatory proteins to the immune system [10], to gene therapy approaches to express antibody-like molecules [11], to the generation of safer versions of live-attenuated virus vaccine candidates [12].
